# Examining mental health correlates of hate-motivated behaviour in Scotland: An investigation of victims, perpetrators and victim-perpetrators

**DOI:** 10.1177/00207640241262732

**Published:** 2024-06-24

**Authors:** Kirsten Russell, Simon C Hunter, Abigail Post, Susan Rasmussen, Robert J Cramer

**Affiliations:** 1 Department of Psychological Sciences and Health, University of Strathclyde, Glasgow, UK; 2 Department of Psychology, Glasgow Caledonian University, UK; 3 Faculty of Education, University of Western Australia, Perth, Australia; 4 Department of Public Health Sciences, University of North Carolina at Charlotte, USA; 5 Violence Prevention Center, University of North Carolina at Charlotte, USA

**Keywords:** Hate crimes, microaggressions, mental health

## Abstract

**Background:**

Hate-motivated behaviour (HMB) ranges from microaggressions to criminal acts and is a public health concern with wide-ranging consequences.

**Aims:**

The current study aimed to examine the mental health correlates of HMB perpetration, victimisation and co-occurring victimisation/perpetration.

**Methods:**

Participants (*n* = 447) completed an online cross-sectional survey assessing demographic factors, HMB (perpetration and victimisation), positive mental wellbeing and symptoms of depression and anxiety.

**Results:**

HMB victimisation was associated with lower positive mental wellbeing and increased symptoms of anxiety and depression. However, neither HMB perpetration nor co-occurring perpetration/victimisation were associated with any of the three mental health outcome measures.

**Conclusion:**

Experiencing HMB as a victim is linked to increased psychological distress. Additional research, which focuses on sampling populations who are known to be at greater risk for involvement in HMB, is needed to fully understand the impact of the victim-offender overlap on mental health outcomes.

## Introduction

Hate-motivated behaviour (HMB), understood as verbal or nonverbal expressions of discrimination ranging from microaggressions to hate crimes (Cramer et al., 2021), is a serious public health problem in Scotland. According to government data collected from 2020 to 2021, there were over 6,000 police-reported hate crimes in Scotland (Scottish Government, 2023). This trend has remained since 2014. HMB victimisation is important to address as it has been associated with far-reaching negative outcomes, including poor mental health among victims (Cramer et al., 2023).

Whilst evidence from the field of criminology consistently supports the robust overlap between victimisation and offending (Jennings et al., 2012), little research has examined mental health in relation to HMB perpetration or in victim-perpetrators. This represents an important gap in our understanding, as studies have suggested those involved in committing criminal behaviour experience poorer health outcomes (Leiding et al., 2022) but are less likely to report their own experiences of victimisation (Berg & Felson, 2018). In turn, victim-perpetrators of HMB may not receive the services they need.

Therefore, the purpose of this study was to investigate the impact of HMB victimisation, perpetration and co-occurring victimisation/perpetration on mental health (e.g. anxiety, depression and mental wellbeing) in a Scottish sample using a recently developed HMB checklist (Cramer et al., 2023). It was hypothesised that experiences of HMB victimisation, perpetration and co-occurring HMB victimisation-perpetration would be associated with greater anxiety, depression and mental wellbeing.

### Procedure

The sample was drawn from a nationwide survey study investigating experiences of HMB. This investigation adhered to the British Psychological Society’s ethical guidelines and approval was obtained from the University Ethics Committee (#UEC21/83). The investigation was conducted online via Qualtrics. To be eligible for participation, participants had to be over the age of 18 years and currently living in Scotland. The study was advertised on online platforms including Twitter and Facebook, and an advert was also placed on a virtual university research recruitment platform. Posters advertising the research were also placed around the campus of one university in Scotland.

Participants were given access to a detailed information sheet. Informed consent was requested before participants were able to access the survey. Participants were then asked to complete a basic demographics questionnaire, followed by a range of measures, presented in a randomised order. The survey took approximately 30 minutes to complete. A downloadable debrief sheet was provided upon completion.

### Participants

Table 1 contains sample demographic information. The sample was predominantly comprised of White Scottish (79.4%), female (79.0%), low income, young adults (average age 23-years old).

**Table 1.** Sample demographic information.

Variable	*n* (%)	*M* (*SD*)
Age	–	22.84 (7.27)
Estimated annual income	–	£13,346.98 (£12,620.58)
Gender		
Male	76 (17.0)	–
Female	353 (79.0)	–
Non-binary	9 (2.0)	–
Transgender female	4 (0.9)	–
Transgender male	2 (0.4)	–
Queer	3 (0.7)	–
National identity		
Scottish	372 (83.2)	–
English	33 (7.4)	–
Northern Irish	8 (1.8)	–
British	3 (0.7)	–
Malaysian	5 (1.1)	–
Irish	4 (0.9)	–
Omani	3 (0.7)	–
Other	18 (4.0)	–
Missing	1 (0.2)	–
Ethnicity/race		
White Scottish	355 (79.4)	–
White Irish	8 (1.8)	–
White other British	29 (6.5)	–
White Polish	2 (0.4)	–
Asian/Asian Scottish/Asian British	21 (4.7)	–
African/African Scottish/African British	2 (0.4)	–
Biracial	3 (0.7)	–
Multiracial	7 (1.6)	–
Other White (e.g. Austrian)	12 (2.7)	–
Other	6 (1.3)	–
Missing	2 (0.4)	–
Sexual orientation		
Gay	10 (2.2)	–
Lesbian	16 (3.6)	–
Heterosexual	300 (67.1)	–
Bisexual	64 (14.3)	–
Queer	15 (3.4)	–
Questioning	6 (1.3)	–
Pansexual	13 (2.9)	–
Asexual	3 (0.7)	–
Prefer no label	19 (4.3)	–
Missing	1 (0.2)	–
Political affiliation		
Scottish national party	194 (43.4)	–
Conservative	10 (2.2)	–
Labour	96 (21.5)	–
Liberal Democrats	16 (3.6)	–
Green Party	57 (12.8)	–
Other (e.g. none)	56 (12.5)	–
Missing	18 (4.0)	–
Religion		
Catholic	98 (21.9)	–
Protestant	49 (11.0)	–
Baptist	1 (0.2)	–
Christian other	20 (4.5)	–
Muslim	10 (2.2)	–
Buddhist	3 (0.7)	–
Atheist	124 (27.7)	–
Agnostic	61 (13.6)	–
Spiritual	24 (5.4)	–
Other (e.g. no religion)	17 (3.6)	–
Prefer not to say	33 (7.4)	–
Missing	7 (1.6)	–

*Note. N* = 447; *M* = mean; *SD* = standard deviation.

### Measures

#### Demographics

Participants were asked to provide information regarding their age, gender identity, ethnicity, sexual orientation, financial status, political affiliations and religious beliefs.

#### Hate-motivated behaviour

History of engaging in HMB was captured using the Hate-Motivated Behaviour Checklist (HMBC; Cramer et al., 2021; α = .88). Participants were asked how often they had engaged in 26 specific HMB (i.e. those driven by actual or perceived group membership) that encompass violence (e.g. hit/punched a person), property crime (e.g. wrote graffiti) and noncriminal micro-aggressive acts (e.g. told jokes).

To assess participants lifetime experiences of self-reported HMB victimisation, we adapted the original checklist to ask whether the participants had experienced the 26 HMBs themselves because of their own demographic characteristics/group membership (see Supplemental Material 1 for this Hate Motivated Behaviour Checklist -Victimisation (HMBC-V) measure)). The HMBC-V displayed excellent internal consistency (α = .92)

#### Depressive and anxiety symptoms

The Hospital Anxiety and Depression scale (HADS) contains two 7-item subscales that assess the extent to which participants have experienced depressive (α = .68) and anxious symptomology (α = .84) in the past week (Zigmond & Snaith, 1983). Total scores for each subscale are calculated by taking a sum of responses.

#### Positive mental wellbeing

The short version Warwick-Edinburgh mental well-being scale (SWEMWBS; Tennant et al., 2007) comprises seven positively worded items that relate to different aspects of positive mental health (α = .88). Higher scores indicate more positive mental well-being.

### Analytical plan

A path analysis was estimated using Mplus Version 7.31 (Muthén & Muthén, 2012) which regressed Depression, Anxiety and Wellbeing on to the HMBC-V, HMBC and an indicator representing the interaction term (HMBC-V*HMBC). The interaction term was created in SPSS by computing the product of the HMBC-V and HMBC. The MLR estimator was implemented in MPlus and utilised Full Implementation Maximum Likelihood (FIML) to address missing data. The three outcome variables were permitted to covary as were the three predictor variables. No fit indices are reported for the path analysis since it was a saturated model. Model syntax is presented in the Supplemental Material.

## Results

### Descriptive statistics

Table 2 displays correlations, means, standard deviations and ranges for the five scale scores subsequently included in the path analysis.

**Table 2.** Means, standard deviations, ranges and correlational coefficients for mental HMBC, HMBC-V, anxiety, depression and mental wellbeing.

	1	2	3	4	5
1. HMBC-V	–	.55***	.39***	40***	−.23***
2. HMBC		–	.16***	.25***	−.07
3. Anxiety			–	.57***	−.63***
4. Depression				–	−.64***
5. Wellbeing					–
*M*	8.25	3.91	10.75	5.55	22.12
*SD*	6.65	4.09	4.24	3.44	5.02
Range	0–26	0–26	0–21	0–18	7-35

*Note*. Listwise deletion was implemented in SPSS28 and so *N* varied from 386 to 442.

*** *p* < 0.01.

### Path analysis

**Figure 1.**
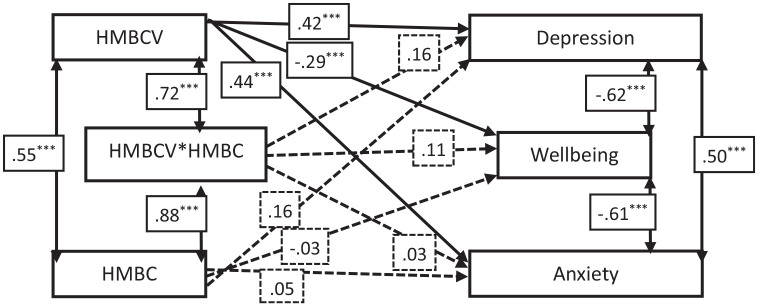
Path analysis results. *Note*. Solid lines indicate significant paths, dashed lines indicate non-significant paths. ***p < .01.

The HMBC-V was significantly and positively associated with both depression and anxiety, and was significantly, negatively associated with wellbeing. Neither HMBC (i.e. perpetration) nor the interaction term were significantly associated with any of the three outcome measures (Figure 1). The model accounted for 15.7% of the variance in depression, 15.2% of the variance in anxiety, and 5.2% of the variance in wellbeing.

## Discussion

This study investigated HMB victimisation and perpetration, as well as their interactions, on mental health. Consistent with prior literature (e.g. Lockwood & Cuevas, 2022; Spanierman et al.., 2021), HMB victimisation was associated with worse mental health. Grounded in criminological theories (e.g. routine activity theory), evidence shows a robust victim-offender relationship across types of criminal behaviour (e.g. Jennings et al., 2012). We found that a moderate positive association in the HMB context; however, the interaction offered no additional value in understanding mental health sequalae linked to experiences of HMB.

Our findings provide initial insight into the study and use of HMB through HMBC and HMBC-V measurement. Cross-cultural research highlights that, at an international level, progress towards a cumulative and comparative evidence base in relation to HMB is limited by variability in definitions, terminology, criteria and disconnected data sources (Sheppard et al., 2021; Vergani et al., 2022). The HMBC was developed in pursuit of a singular measurement tool that could overcome these limitations. Following on from prior HMBC research (Cramer et al., 2023), the perpetration instrument appears appropriate for adaptation to a victimisation format, as indicated by acceptable in internal consistent and expected associations with mental health. With that said, future work employing both HMBC versions can elaborate on the examination of the victim-offender overlap of HMB in relation to other criminogenic samples (e.g. adjudicated offenders) and outcomes (e.g. recidivism). Study limitations include a cross-sectional, online survey design and procedure, as well as a restricted sample with respect to age and race. Additional HMB research may employ community-engaged approaches to obtain more diverse samples in Scotland.

## Supplemental Material

sj-docx-1-isp-10.1177_00207640241262732 – Supplemental material for Examining mental health correlates of hate-motivated behaviour in Scotland: An investigation of victims, perpetrators and victim-perpetrators Supplemental material, sj-docx-1-isp-10.1177_00207640241262732 for Examining mental health correlates of hate-motivated behaviour in Scotland: An investigation of victims, perpetrators and victim-perpetrators by Kirsten Russell, Simon C Hunter, Abigail Post, Susan Rasmussen and Robert J Cramer in International Journal of Social Psychiatry
