# Supplementary material for: Examining mental health correlates of hate-motivated behaviour in Scotland: An investigation of victims, perpetrators and victim-perpetrators
Source: Int J Soc Psychiatry. 2024 Jun 24;70(7):1341–5. doi: 10.1177/00207640241262732 (PMC11528929; doi:10.1177/00207640241262732)
Supplement: sj-docx-1-isp-10.1177_00207640241262732 – Supplemental material for Examining mental health correlates of hate-motivated behaviour in Scotland: An investigation of victims, perpetrators and victim-perpetrators [file sj-docx-1-isp-10.1177_00207640241262732.docx]

**Online supplement**

**Supplement 1**

HMBC-V Section I: Behaviours

Instructions: Using the scale below, please tell us if you have experienced the following actions (in your lifetime) as a result of another person knowing or believing they know your demographic characteristics (i.e., race, ethnicity, gender, sexual orientation, religion, disability, or national origin). That is, we are interested in the extent to which you have experienced these behaviours from others because of your race, ethnicity, gender, sexual orientation, religion, disability, and so on.

0 = No 1 = Yes

No Yes

1. Slurs or belittling name-calling:

2. Jokes at your expense:

3. Verbally threatened:

4. Been followed:

5. Received repeated, unwanted emails:

6. Received repeated, unwanted text messages:

7. Received repeated, unwanted phone calls/voice messages:

8. Been spat at:

9. Had insults yelled at you (other than slurs or belittling name-calling):

10. Had objects thrown at you:

11. Had your property damaged (e.g., technology, mailbox, home):

12. Been hit/punched:

13. Been pushed/shoved:

14. Been hit with object (e.g., bat, stick):

15. Been physically fought with (e.g., prolonged encounter using fists, slapping, kicking, tripping):

16. Someone attempted to physically fight you:

17. Unwanted sexual contact (e.g., touching, grinding):

18. Forced sex:

19. Attempted forced sex:

20. Been stolen from:

21. Had graffiti targeted at you:

22. Been stared or scowled at:

23. Been the target of negative talk (e.g., rumors, gossip, defaming):

24. Received hostile posts on your person’s social media page (e.g., Facebook, Twitter):

25. Been forced to behave a certain way (i.e., bullying):

26. Had someone avoid interpersonal contact with you:

27. Other actions (Please specify the other behaviours that you have experienced): ______________________________________________________________________________________________________________________________________________________
